# Access to the Lumbosacral Spine: A Current View

**DOI:** 10.1055/s-0044-1785462

**Published:** 2024-04-10

**Authors:** Allan Hiroshi de Araujo Ono, Aécio Rubens Dias Pereira Filho, Fernando Flores de Araujo, Raphael Marthus Marcon, Alexandre Fogaça Cristante, Tarcisio Eloy de Pessoa Barros Filho

**Affiliations:** 1 Instituto de Ortopedia e Traumatologia, Hospital das Clínicas da Faculdade de Medicina, Universidade de São Paulo, São Paulo, Brasil; 2 Hospital Vera Cruz, Campinas, SP, Brasil; 3 Departamento de Ortopedia e Traumatologia, Faculdade de Medicina, Universidade de São Paulo, São Paulo, Brasil

**Keywords:** arthrodesis, lumbosacral region/surgery, spine/surgery, spinal fusion

## Abstract

The surgical approach to the lumbosacral spine has been the subject of experimental and scientific anatomical studies since the Hippocratic era. However, it was in the 20th century that, with the evolution of asepsis and antibiotic therapy, spine surgery began to evolve at breakneck speed, and the various possibilities of access roads became objects of development and discussion. As a result, pathologies of the lumbosacral spine can be accessed in different ways and positions, from the traditional posterior approach in the prone position to the anterior, oblique, lateral, and endoscopic approaches. The current article brings state-of-the-art access routes to the lumbosacral spine. This article objective is to elucidate the possibilities of accesses the lumbar spine for any purposes, as decompression, fusion, tumour resections, reconstruction or deformity correction, despites type of implants or implants positioning.

## Introduction

Spine surgery is one of the areas with the most remarkable development among surgical specialties concerning the technologies employed in the equipment for obtaining images, navigators, implants, and materials, as well as the surgical techniques and access routes. Less traumatic retractors, tubes, and endoscopes have evolved, thus providing increasingly less invasive accesses and promising results. 1 Spinal fusion surgery was described for the first time in 1911 by Hibbs, who described the technique of decortication and morselization of the autologous graft in a case of chronic osteomyelitis caused by Pott's disease. 2

In 1933, Burns and Capener found the possibility of approaching the spondylolisthesis of L5-S1 through the anterior route after several cadaveric studies. 3 They performed the surgery on a 14-year-old boy, performing anterior route access to the region of the retroperitoneum with a left paramedian incision and transperitoneal access where they accessed the spine, placing an autologous graft. After two months of plastered immobilization, the boy recovered. 4

The first descriptions of percutaneous intradiscal procedures were described in the 1960s by Lyman Smith, such as chemonucleolysis with papain, shortly after Hijikata described the possibility of removing the nucleus pulposus percutaneously with the aid of small tubes and a pituitary tweezers using the same parameters that he used to perform his discographies. 5

In 1986 Kambin described the safe anatomical corridor to access the intervertebral disc, between the nerve root and the superior facet, receiving the name "Kambin's triangle" his pioneering work allowed the development of the first endoscopic surgeries. 5

**Fig. 1:**
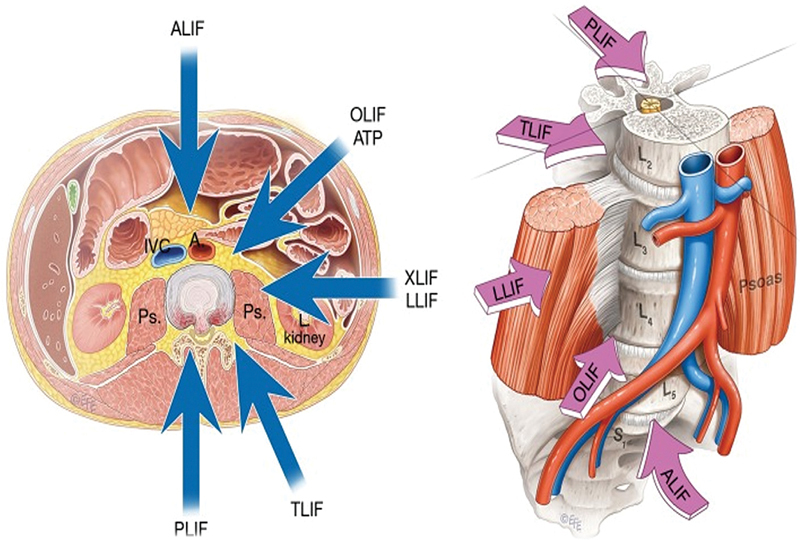
Possible approaches to lumbar intervertebral fusion.

The different accesses to the lumbar and lumbosacral spine allowed us a significant evolution in surgical techniques and methods, as well as the development of technologies for the resolution of the main pathologies, which continue to be objects of discussions and studie 6 ( Fig. 1 ).

## Posterior Approach

The posterior access routes to the vertebral column allow a direct approach to the vertebral canal without manipulating larger vascular or neural structures. They are, therefore, the first to be discovered by ancient surgeons, despite little anatomical knowledge and frank discouragement of the surgical approach.

The posterior access to the spine appears in some descriptions of antiquity, mainly intending to remove fragments of fractures from the vertebral canal. However, the lack of knowledge of antisepsis and hemostasis only allowed the flourishing of spinal surgery from the 19th century onwards. 7

We consider the median subperiosteal and paravertebral options when considering the posterior approaches. For this, it is essential to know the posterior muscular anatomy of the lumbosacral spine, which is formed by the following muscles: Multifidus (M), Longissimus (L), and Iliocostalis (Il).

The multifidus is the largest and most medial posterior muscle, poly segmental and multiplies innervated. It has intimate contact with the lamina and the spinous and transverse processes and is considered the most critical posterior stabilizer of the lumbosacral, commonly presenting itself atrophic in lumbar degenerative pathologies. 8

The three muscles run longitudinally along the spine, inserting into the sacrum, the sacroiliac joint, and the iliac wing, but the multifidus has a unique short, robust shape. Its architecture allows the creation of large forces in short distances, producing more forces with the spine in anterior flexion, protecting it in its most vulnerable position. 8

**Posterior median approach** : in this access route, an incision is made in the skin over the spinous processes, continued by the subperiosteal bone dissection with the detachment of the multifidus muscle from the spinous processes, laminae and reaching the region of the transverse processes using Cobb instruments and electrocautery, being able to offer complete exposure of the posterior bone elements of the spine, allowing extensive decompression surgeries through a laminectomy, resection of bone or intracanal tumours, in addition to allowing posterolateral and inter somatic fusion through the PLIF (posterior inter somatic fusion) and TLIF (transforaminal inter somatic fusion) techniques, in addition to surgical correction of scoliosis. 9

The disadvantage of this method is the surgical aggression to the paraspinal musculature, which leads to postoperative muscular atrophy, mainly of the multifidus muscle, reducing up to 27% of its area below the arthrodesis site, which may lead to less satisfactory surgical results, when compared to with techniques that preserve more posterior musculature. 10 11

The mechanisms by which muscle injuries occur are dissection, which tears the tendon insertions, and the excessive use of electrocautery, causing thermal injury and tissue necrosis. However, the most associated factor is the use of self-static retractors for prolonged periods. The degree of injury is linked to the time of retraction, and the intermittent loosening of the retractors reduces the area of muscular injury. 12 13

**Paraspinal Approach** : This approach uses the dissection of the intermuscular plane between the multifidus muscle and the longissimus muscle, described by Wiltse in 1968 as a modification of the approach described by Watkins, used an approach between the sacrospinatus muscle and the quadratus lumborum. 14 15

This access has a lower rate of bleeding and less muscle destruction, in addition to allowing direct access to the transverse processes, pedicle, and intervertebral discs, being advantageous in approaching mainly hernias in the region of the vertebral recess, foraminal and extraforaminal. 16

The access can be performed as the original description with two paramedian incisions or a single central incision and two in the subfascial plane, as modified by Wiltse himself in 1988. 17 However, the original technique with two incisions seems more advantageous with a lower rate of complications such as suture dehiscence and seroma. 16 This access allows several surgical approaches, with TLIF transforaminal inter somatic fusion being one of the most popular, as it is a less invasive approach par excellence. Compared with the traditional media approach, it has several advantages, such as less postoperative muscle atrophy, lower incidence of adjacent degeneration level, lower infection rate, and less intraoperative bleeding. 18 19

## Lateral Access

The lateral approach to the spine was first described by Ozgur et al. 20 as a less invasive alternative to vertebral inter somatic fusion, and its use has become popular in the treatment of a variety of pathologies of the spine, more recently being adapted for the L5-S1 use in an anterolateral approach, anterior to the iliopsoas muscle, also called the iliopsoas access oblique. 21 22

According to the original description of the technique, the positioning is in lateral decubitus on an inverted radiolucent table, with the greater trochanter positioned on the fold of the table, placed in an orthogonal position with the radioscopy. The table is flexed to facilitate the dissection by moving away the ribs of the iliac crest. The skin incision should be made towards the disc for one level or towards the vertebral body for two levels; for three levels or more, more than one incision is required, the dissection of the external oblique, internal oblique, and transversus abdominis muscle, it must be done bluntly, without the use of electrocautery. In order to avoid injury to peritoneal structures, the index finger must clear the retroperitoneal space before inserting the guide wire, dilators, and retractor that cross the iliopsoas directly with the aid of a magnifying glass or microscope. This approach is used for lateral intersomatic fusion called LLIF (Lateral Lumbar Intersomatic Fusion) or XLIF (Extreme Lateral Intersomatic Fusion), they are synonyms , but XLIF were patented by a company as the implant name too, so is more correct to use LLIF for the Lateral approach of intersomatic fusion.

Multimodal neurophysiological monitoring is mandatory to avoid lumbar plexus and genitofemoral nerve injuries. 23 Knowledge of the regional neuroanatomy, the time of retraction of the iliopsoas muscle, and appropriate training in the technique allow its performance without needing an access surgeon. 24

## Anterior Access

Anterior approaches to the lumbar spine (ALIF) first emerged in the 1930s for the treatment of spondylolisthesis by Capener 3 and Pott's disease by Ito et al. 25

Since then, various surgical techniques, including open or laparoscopic transperitoneal approaches and retroperitoneal exposures, have been developed. Since the end of the 1990s, the preferred and most used surgical technique has been the minimally invasive, popularized by Brau 26 The ALIF approach provides direct midline exposure of the lumbar disc, thereby allowing for a wide discectomy and placement of a sizeable interbody graft that maximizes coverage of the vertebral plateau. L5-S1 is the preferred level of treatment as it avoids the complexity of the aorta/caval/iliac bifurcation. However, higher levels from L2 to S1 have already been performed by experienced surgeons. 27

The anterior access technique to the lumbosacral spine is helpful in degenerative disc disease, isthmic or degenerative spondylolisthesis, spondylodiscitis, pseudarthrosis, removal of mispositioned or migrated TLIF or PLIF cages, disease, or degeneration of the adjacent level. 28

The posterior approach has a higher risk of neurological damage and dural tear due the presence of fibrosis, with the anterior access being preferred and with less morbidity. 27 Restoration of sagittal balance allows using hyper lordotic implants (up to 30 degrees) and the desired rebalancing of vertebral body injuries from fractures or tumours.

Some advantages of this technique include the direct view of the midline of the disc space and the extensive lateral exposure of the vertebral bodies, which allows an efficient release of the disc space, adequate access to the entire ventral surface of the disc exposed, allowing complete discectomy, restoration of disc space, automatically reducing deformity through ligamentotaxis, leading to indirect decompression. It allows the sparing of the spine and muscles anterolateral posterior muscles to the psoas, which can reduce postoperative pain and disability. 6 27 28

Disadvantages of the ALIF technique include approach-related complications such as vascular injuries, retrograde ejaculation, and visceral injuries. 29 30 The ALIF technique is suitable for the L4/L5 and L5/S1 levels due to the vascular anatomy that provides a large working corridor between the iliac vessels. Higher levels, such as L2/3 and L3/L4, can be explored by teams with experienced and trained access surgeons due to the need for extensive vascular mobilization of the aorta and iliac arteries and retroperitoneal viscera, such as the pancreas and kidneys. 6

The contraindication for any anterior approach includes previous major abdominal surgeries, severe peripheral vascular disease, patients with a history of radiotherapy, stents, and endoprostheses of abdominal vessels. In addition, specific contraindications include the need for direct posterior decompression, acute infection, severe osteoporosis due to the risk of subsidence (fracture and sinking of the plateau), extruded disc herniation, migrated, especially if calcified, requiring posterior approach. 6 31 Obesity is a relative contraindication showing similar intraoperative morbidity, postoperative complications, and arthrodesis similar to patients with BMI within the normal range. 32

## Oblique Access

The first description of an OLIF approach was published in 1997 by Mayer. 33 However, the official name and acronym were not coined until 2012 when Silvestre et al. 34 used a minimally invasive retroperitoneal approach similar to the Mayer approach for anterior lumbar intervertebral fusion. This technique is reported by Silvestre et al. 34 as OLIF and accesses the anterolateral surface of the disc space before the psoas muscle, which is mobilized posteriorly. After preparing the disc space, the inter somatic device is inserted at an oblique angle and rotated in a lateral position.

The evolution of technique and instrumentation provided adequate access to the lumbar spine, with low rates of perioperative complications, short surgical times and low morbidity and mortality, little postoperative pain, and early return to daily activities. Indications include degenerative disc diseases, discogenic lumbar pain, degenerative lumbar scoliosis, low-grade spondylolisthesis, lumbar instability, revision, mild to moderate canal stenosis, sagittal deformity, fractures, tumours, adjacent level disease. 28

The oblique device can raise the intervertebral space's height and expand the intervertebral foramen's size, indirectly achieving decompression. 35 Regarding anatomical accessibility, the OLIF technique can reach L1 to S1 using an oblique corridor between the aorta, inferior vena cava, and psoas muscles to access the disc space.

There is adequate operating space with the left oblique corridor from levels L2 to L5, with the additional ability to be extended during surgery with lateral positioning and retraction of the psoas muscle. The ribcage can limit access at the level of L1-L2 and the iliac crest and iliac vessels at the level of L4-L5. 27 31

The advantages are less invasive operative technique, reduced operative time, and less chance of injury to the lumbar plexus, allowing direct access to the disc. Without injury to the lamina, it leads to less damage and bleeding, lower rate of nerve injury, faster postoperative recovery, less pain, short hospital stay, and increased rates of inter somatic fusion caused by a large amount of disc removal, promoting a large area of contact with the plateaus, and suitable correction of the deformities. 6 36 37

There is also the possibility of approaching greater levels through the same access without enlarging the operative window. Disadvantages include anatomical alterations that limit the procedure and the potential risks involved, such as sympathetic dysfunction and vascular injury. 21 31

We can observe that in the aorta/cava/high iliac bifurcations (those that occur above the upper 1/3 of L-4), the iliac vessels are already well open and with an almost lateral trajectory at the level of the L4-5 disc, the which would lead to vascular damage in the supposed OLIF access corridor.

Considering the report above, in positioning the lower blade of the retractor/retractor, we must avoid its fixation in the vertebra of L-5 with the fixation screw. 6 21 31

There are several limitations to the oblique access approach.

There is the risk of canal stenosis caused by the inter-somatic device in the posteromedial trajectory, leading to the disc or ligament material displacement towards the central canal or the contralateral foramen. A second limitation relates to patients with high-grade spondylolisthesis because there is not enough "overlapping" of the two vertebral plateaus to accommodate the inter somatic device in an oblique trajectory.

The procedure may also be affected in congenital vertebral canal stenosis and for injuries that occupy the intervertebral space, in a spontaneous fusion of intervertebral space or posterior facets, and by the shape of the psoas muscle. Therefore, on the concave side of scoliosis patients, the space between the vessels and the psoas muscle decreases, which is not conducive to the OLIF pathway.

Psoas sign interiorized, that is, on axial images at the level of L4-L5, the psoas muscle in the area of entry into the lateral annulus of the vertebral disc. The space between the psoas muscle and the quadratus lumborum muscle increases in some patients, which could lead to misunderstanding the distance gap between the artery and the psoas muscle. The different positions influence the shape of the psoas muscle. In the right lateral decubitus position, the left psoas major muscle is affected by gravity and is close to the vertebral body. 6 31 38

## Endoscopic Approach

For a spinal approach to be considered a fully endoscopic surgery, it needs to meet the following criteria: the use of an endoscope that has a working channel and endoscopic system, an utterly percutaneous approach with a small incision like a skin puncture ("stab incision"), a single-portal technique performed under constant saline irrigation. 39 40

**Fig. 2:**
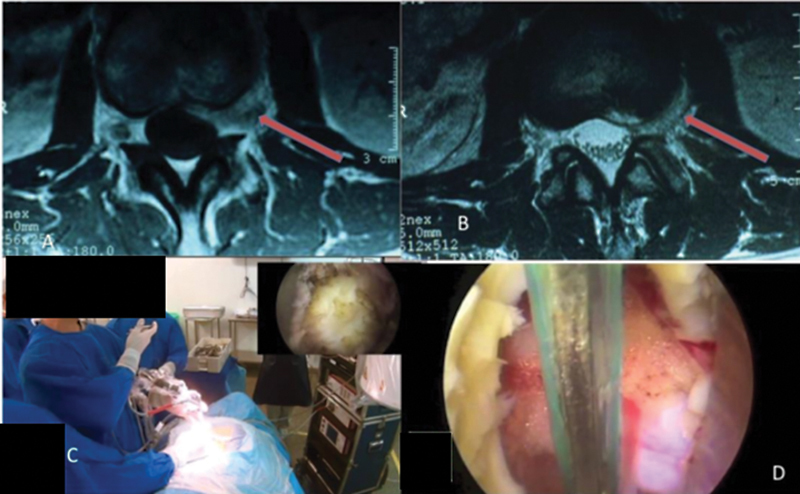
Transforaminal endoscopic approach. (A) location of the nerve root ganglion, (B) direct access to the disc abscess, (C) positioning of the team, (D) endoscopic view of the neural root.

There are other methods of endoscopic approach to the spine, such as bi-portal endoscopy, microendoscopy, epiduroscopy, and video-assisted tubular surgery. However, endoscopic or completely endoscopic percutaneous spine surgery ( Fig. 2 ) is the most commonly used in our setting. 39 40

When considering the lumbar spine, the main endoscopic access routes can be divided into the transforaminal and interlaminar approaches. 41 42

## Transforaminal Approach

It can be performed with the patient in a prone position with flexion of the hips and knees under local anesthesia, sedation, or general anesthesia. In the latter case, it may be accompanied by neurophysiological monitoring to allow the surgeon to monitor the function of the emerging root and passer-by of the approached level. 41 42

The principles of the endoscopic transforaminal route are to seek access as close as possible to the disc pathology and to avoid irritation of the emerging root. To achieve these principles, the foraminoplasty can be associated with the procedure, which is the recalibration of the approached intervertebral foramen using ways to remove or thin the bone edges. 42 43 This can be done with shaver burs, foraminoplasty burs, or percutaneous refers.

The angle of access and the access point in the foramen is essential for the technique's success. They can be adjusted according to the patient's body size, disc level, and pathology site to be approached. Generally, at lower lumbar levels (L4-L5 and L5-S1), a distance from the midline of 10-12 cm is used, and at higher levels, a progressively closer approach to the midline is used due to renal topography.

After a posterolateral incision, a guide wire is inserted, seeking to reach the space of Kambin's triangle. This approach safety triangle is defined as the space between the laterally emerging root, medially dura mater, and the base, the superior portion of the pedicle of the inferior vertebra.

The access route is dilated with progressive blunt dilators until the working sleeve is placed. After the work jacket is securely positioned, the endoscope is positioned. The ideal way to guarantee the correct positioning is to adequately visualize the structures that make up the intervertebral foramen to allow a safe surgery. 42 43

There are some divergences in the literature regarding the disc approach. Initially, the transforaminal endoscopic approach was performed from the central portion of the disc and progressed towards the outer part of the disc, a technique known as inside-out.

With the evolution of surgical techniques and equipment technologies for disc pathologies emerged the technique of approaching from the outside, known as outside-in. 42 43 44

More recently, with the transforaminal approach to approach bone pathologies of the foramen, without the need to approach the disc, the outside-out technique corresponds to techniques of bone decompression of the foramen. This approach offers risk of residual pain due root manipulation of the procedure, especially during surgeons learning curve, even so neurologic complications are lower in percutaneous endoscopic techniques when compared to open approach. 45

## Interlaminar Approach

Like transforaminal endoscopy, it can be performed under sedation or local or general anesthesia. The approach's target point is the interlaminar window's lateral edge. 41 43 This window is usually more prominent at more caudal levels, progressively decreasing in the cephalad direction. Thus, the L5-S1 is the level with the largest interlaminar window. Initially, the interlaminar access route required good-sized windows to ensure a safe approach.

The evolution of the surgical technique and bone decompression equipment made it possible to approach even smaller interlaminar windows after adequate bone decompression. 43 46 In the interlaminar approach, the guide wire can be used initially or even the direct approach with the blunt dilator due to the risk of inadvertent introduction of the guide wire into the vertebral canal through the ligamentum flavum.

After the dilator, the working cannula is positioned with the bevel resting on the most lateral portion of the window. Next, the endoscope is introduced to ensure visualization of the ligamentum flavum. 41 46 This can be removed using appropriate tweezers and scissors, allowing visualization of the epidural space.

After the flavectomy, the surgical field is prepared with more effective bone decompression and/or soft tissue removal until it is possible to visualize and dissect the neural structures. 41 46 Through this route, it is possible to treat disc or bone compressive pathology, depending on the origin of the symptoms. At the end of the surgery, decompression can be confirmed with a blunt probe used through the working channel and visualization of free and pulsatile structures with the serum flow. 41 46

## Final Considerations

The various access routes and surgical approaches described here continue to be objects of study, generating dozens of publications annually. There is no consensus on the best approach for each pathology. However, a trend seeks a reduction in tissue damage, especially muscle, effectiveness in the decompression of neural structures, and greater safety with minimization of the risks of neurological damage and postoperative infections. In addition, advances in imaging and magnification techniques allow smaller, more effective, and safer accesses.
