# Supplementary material for: Access to the Lumbosacral Spine: A Current View
Source: Rev Bras Ortop (Sao Paulo). 2024 Apr 10;59(2):e153–9. doi: 10.1055/s-0044-1785462 (PMC11006527; doi:10.1055/s-0044-1785462)
Supplement: Related texts

# Research article: Acesso à coluna lombossacral: Visão atual

**Keywords:** artrodese, coluna vertebral/cirurgia, fusão vertebral, região lombossacral

## Abstract

A abordagem cirúrgica da coluna lombossacral tem sido objeto de estudos anatômicos experimentais e científicos desde a era hipocrática. Contudo, foi no século XX que, com a evolução da assepsia e da antibioticoterapia, a cirurgia da coluna começou a evoluir em velocidade vertiginosa e as diversas possibilidades de vias de acesso tornaram-se objetos de desenvolvimento e discussão. Desta forma, as doenças da coluna lombossacral podem ser acessadas de diferentes maneiras e posições, desde a abordagem posterior tradicional em decúbito ventral até as abordagens anterior, oblíqua, lateral e endoscópica. O presente artigo traz vias de acesso de última geração para a coluna lombossacral. O objetivo deste artigo é elucidar as possibilidades de acesso à coluna lombar para quaisquer finalidades, como descompressão, fusão, ressecções tumorais, reconstrução ou correção de deformidades, independentemente do tipo de implante ou seu posicionamento.

## Introdução

A cirurgia de coluna é uma das áreas de maior desenvolvimento dentre as especialidades cirúrgicas em referências às tecnologias empregadas em equipamentos para obtenção de imagens, navegadores, implantes, materiais, técnicas cirúrgicas e vias de acesso. A evolução de afastadores, tubos e endoscópios menos traumáticos propicia acessos cada vez menos invasivos e resultados promissores. 1 A cirurgia de fusão espinhal foi descrita pela primeira vez em 1911 por Hibbs como uma técnica de decorticação e morselização de enxerto autólogo em um caso de osteomielite crônica decorrente da doença de Pott. 2

Em 1933, Burns e Capener perceberam a possibilidade de abordagem da espondilolistese de L5-S1 por via anterior após vários estudos cadavéricos. 3 A cirurgia foi realizada em um menino de 14 anos por meio de acesso anterior à região do retroperitônio, com incisão paramediana esquerda e abordagem transperitoneal, onde acessaram a coluna vertebral e implantaram um enxerto autólogo. Após dois meses de imobilização gessada, o menino se recuperou. 4

As primeiras descrições de procedimentos intradiscais percutâneos são da década de 1960 e foram feitas por Lyman Smith, como a quimionucleólise com papaína, logo após Hijikata descrever a possibilidade de remoção percutânea do núcleo pulposo com auxílio de pequenos tubos e uma pinça hipofisária utilizando os mesmos parâmetros que costumava apresentar suas discografias. 5

Em 1986, Kambin descreveu o corredor anatômico seguro para acesso ao disco intervertebral, entre a raiz nervosa e a faceta superior, que recebeu o nome de “triângulo de Kambin”. Seu trabalho pioneiro permitiu o desenvolvimento das primeiras cirurgias endoscópicas. 5

Os diferentes acessos à coluna lombar e lombossacral permitiram a evolução significativa das técnicas e métodos cirúrgicos, bem como o desenvolvimento de tecnologias para a resolução das principais doenças que ainda são objetos de discussões e estudos 6 ( Fig. 1 ).

**Fig. 1:** Possíveis abordagens à fusão intervertebral lombar. ALIF, fusão intersomática lombar anterior; OLIF, fusão intersomática lombar lateral oblíqua; ATP, abordagem anterior ao psoas; XLIF, fusão intersomática lateral extrema; LLIF, fusão intersomática lombar lateral; VCI, veia cava inferior; PLIF, fusão intersomática posterior; TLIF, fusão intersomática transforaminal.

## Abordagem Posterior

As vias de abordagem posterior à coluna vertebral permitem uma abordagem direta ao canal vertebral sem manipulação de estruturas vasculares ou nervosas maiores. São, portanto, as primeiras a serem descobertas pelos antigos cirurgiões, apesar do pouco conhecimento anatômico e do franco desencorajamento da abordagem cirúrgica.

O abordagem posterior à coluna vertebral aparece em algumas descrições da antiguidade, principalmente com a intenção de retirar fragmentos de fraturas do canal vertebral. Porém, o desconhecimento da antissepsia e da hemostasia só permitiu o florescimento da cirurgia da coluna vertebral a partir do século XIX. 7

Dentre as abordagens posteriores, estão as opções subperiósteas medianas e paravertebrais. Para isso, é fundamental conhecer a anatomia muscular posterior da coluna lombossacral, formada pelos seguintes músculos: multífido (M), longuíssimo (L) e iliocostal (Il).

O multífido é o maior e mais medial músculo posterior; é polissegmentar e sua inervação é múltipla. Está em contato íntimo com a lâmina e os processos espinhosos e transversos e é considerado o mais importante estabilizador posterior da região lombossacral, comumente apresentando atrofia nas doenças degenerativas lombares. 8

Os três músculos correm longitudinalmente ao longo da coluna, inserindo-se no sacro, na articulação sacroilíaca e na asa ilíaca. O multífido, porém, tem formato único, curto e robusto. Sua arquitetura permite a criação de grandes forças em curtas distâncias, produzindo mais forças com a coluna em flexão anterior, protegendo-a na sua posição de maior vulnerabilidade. 8

**Acesso mediano posterior:** nesta via de acesso, é feita uma incisão cutânea sobre os processos espinhosos, que continua pela dissecção óssea subperióstea com o descolamento do músculo multífido dos processos espinhosos e lâminas até atingir a região dos processos transversos com instrumentos de Cobb e eletrocauterização. Assim, pode oferecer exposição completa dos elementos ósseos posteriores da coluna, permitindo extensas cirurgias de descompressão por meio de laminectomia, ressecção de tumores ósseos ou intracanais. Também permite a fusão posterolateral e intersomática por meio da fusão intersomática posterior (PLIF, do inglês *posterior intersomatic fusion* ) e fusão intersomática transforaminal (TLIF, do inglês *transforaminal intersomatic fusion* ), além de correção cirúrgica de escoliose. 9

A desvantagem desse método é a agressão cirúrgica à musculatura paraespinhal, o que leva à atrofia muscular pós-operatória. Essa atrofia acomete principalmente o músculo multífido, que tem sua área reduzida em até 27% abaixo do sítio da artrodese. Tal atrofia pode levar a resultados cirúrgicos menos satisfatórios em comparação a técnicas que permitem a maior preservação da musculatura posterior. 10 11

Os mecanismos das lesões musculares são a dissecção, que rompe as inserções tendinosas, e o uso excessivo de eletrocautério, o que causa lesão térmica e necrose tecidual. Porém, o fator mais associado é o uso de afastadores autoestáticos por períodos prolongados. O grau da lesão é relacionado ao tempo de retração; além disso, o afrouxamento intermitente dos afastadores reduz a área de lesão muscular. 12 13

**Abordagem paraespinhal:** esta abordagem utiliza a dissecção do plano intermuscular entre os músculos multífido e longuíssimo. Foi descrita por Wiltse em 1968 como uma modificação da abordagem de Watkins entre os músculos sacroespinhal e quadrado lombar. 14 15

Esse acesso apresenta menor taxa de sangramento e menor destruição muscular, além de permitir a abordagem direta aos processos transversos, aos pedículos e aos discos intervertebrais. Desta forma, é indicado principalmente para abordagem de hérnias na região do recesso vertebral, foraminal e extraforaminal. 16

O acesso pode ser realizado de acordo com a descrição original, com duas incisões paramedianas, ou seguindo a modificação proposta pelo próprio Wiltse em 1988, com uma única incisão central e duas incisões no plano subfascial. 17 Porém, a técnica original com duas incisões parece mais vantajosa por apresentar menor índice de complicações, como deiscência de sutura e seroma. 16 Esse acesso permite diversas abordagens cirúrgicas, em especial TLIF, por ser menos invasiva por excelência. Comparada à abordagem medial tradicional, tem diversas vantagens, como menor atrofia muscular pós-operatória, menor incidência de degeneração do nível adjacente, menor taxa de infecção e menor sangramento intraoperatório. 18 19

## Acesso Lateral

A abordagem lateral da coluna foi descrita pela primeira vez por Ozgur et al. 20 como uma alternativa menos invasiva à fusão intersomática vertebral. Seu uso foi rapidamente popularizado no tratamento de diversas doenças da coluna, sendo mais recentemente adaptado para a L5-S1 na abordagem anterolateral, anterior ao músculo iliopsoas, também chamado de acesso oblíquo do iliopsoas. 21 22

De acordo com a descrição original da técnica, o posicionamento é em decúbito lateral sobre mesa radiotransparente invertida, com o trocanter maior na dobra da mesa, ortogonal à radioscopia. A mesa é flexionada para facilitar a dissecção, afastando as costelas da crista ilíaca. A incisão cutânea deve ser feita em direção ao disco em um nível ou em direção ao corpo vertebral em dois níveis; a abordagem a três níveis ou mais exige mais de uma incisão. A dissecção dos músculos oblíquo externo, oblíquo interno e transverso do abdome deve ser feita de forma romba, sem uso de eletrocautério. Para evitar lesões nas estruturas peritoneais, o dedo indicador deve desobstruir o espaço retroperitoneal antes da inserção do fio-guia, dos dilatadores e do afastador, que atravessam o iliopsoas diretamente com o auxílio de uma lupa ou microscópio. Esta abordagem é usada para a fusão intersomática lombar lateral (LLIF, do inglês *lateral lumbar intersomatic fusion* ) ou fusão intersomática lateral extrema (XLIF, do inglês *extreme lateral intersomatic fusion* ). Estes termos são sinônimos, mas XLIF foi patenteado por uma empresa também como nome do implante; portanto, é mais correto usar LLIF para a abordagem lateral da fusão intersomática.

O monitoramento neurofisiológico multimodal é obrigatório para evitar lesões do plexo lombar e do nervo genitofemoral. 23 O conhecimento da neuroanatomia regional, o tempo de retração do músculo iliopsoas e o treinamento adequado da técnica permitem sua realização sem necessidade de cirurgião de acesso. 24

## Acesso Anterior

As abordagens anteriores da coluna lombar, ou seja, a fusão intersomática lombar anterior (ALIF, do inglês *anterior lumbar interbody fusion* ) surgiram pela primeira vez na década de 1930 para o tratamento da espondilolistese por Capener 3 e da doença de Pott por Ito et al. 25

Desde então, várias técnicas cirúrgicas foram desenvolvidas, incluindo abordagens transperitoneais abertas ou laparoscópicas e exposições retroperitoneais. Desde o final da década de 1990, a técnica cirúrgica preferida e mais utilizada tem sido a minimamente invasiva, popularizada por Brau 26 . A abordagem ALIF proporciona exposição direta da linha média do disco lombar, permitindo a discectomia ampla e a colocação de um enxerto intersomático de tamanho considerável que maximiza cobertura do planalto vertebral. L5-S1 é o nível de tratamento preferido, pois evita a complexidade da bifurcação da aorta e das veias cava e ilíaca. Entretanto, níveis mais elevados, de L2 a S1, já foram abordados por cirurgiões experientes. 27

A técnica de acesso anterior à coluna lombossacral é útil na doença degenerativa do disco, espondilolistese ístmica ou degenerativa, espondilodiscite, pseudoartrose, remoção de gaiolas, TLIF ou PLIF mal posicionadas ou que sofreram migração e doença ou degeneração do nível adjacente. 28

A abordagem posterior apresenta maior risco de dano neurológico e ruptura dural devido à presença de fibrose; assim, a via anterior é preferida por ter menor morbidade. 27 O restauro do equilíbrio sagital permite o uso de implantes hiperlordóticos (até 30 graus) e o reequilíbrio desejado de lesões do corpo vertebral por fraturas ou tumores.

Algumas vantagens desta técnica são a visualização direta da linha média do espaço discal e a extensa exposição lateral dos corpos vertebrais, o que permite a liberação eficiente do espaço discal e o acesso adequado a toda a superfície ventral exposta do disco. Isto possibilita a discectomia completa, a restauração do espaço discal e a redução automática da deformidade por meio da ligamentotaxia, levando à descompressão indireta. Além disso, protege a coluna e os músculos anterolaterais posteriores ao psoas, o que pode reduzir a dor e a incapacidade pós-operatória. 6 27 28

Dentre as desvantagens da técnica ALIF, estão as complicações relacionadas à abordagem, como lesões vasculares, ejaculação retrógrada e lesões viscerais. 29 30 A técnica ALIF é adequada para os níveis L4/L5 e L5/S1 devido à anatomia vascular que proporciona um grande corredor de trabalho entre os vasos ilíacos. Níveis mais elevados, como L2/3 e L3/L4, podem ser explorados por equipes com cirurgiões de acesso experientes e com maior treinamento devido à necessidade de ampla mobilização vascular da aorta e artérias ilíacas e vísceras retroperitoneais, como pâncreas e rins. 6

A contraindicação para qualquer abordagem anterior inclui grandes cirurgias abdominais prévias, doença vascular periférica grave, histórico de radioterapia e presença de stents e endopróteses em vasos abdominais. Além disso, dentre as contraindicações específicas, estão a necessidade de descompressão posterior direta, infecção aguda, osteoporose grave pelo risco de subsidência (fratura e afundamento do platô) e hérnia de disco com extrusão ou migração, principalmente se calcificada, necessitando de abordagem posterior. 6 31 A obesidade é uma contraindicação relativa, com morbidade intraoperatória, complicações pós-operatórias e artrodese semelhantes aos pacientes com índice de massa corporal dentro da normalidade. 32

## Acesso Oblíquo

A primeira descrição da abordagem de fusão intersomática lombar lateral oblíqua (OLIF, do inglês *oblique lateral lumbar interbody fusion* ) foi publicada em 1997 por Mayer. 33 No entanto, o nome oficial e a sigla não foram cunhados até 2012, quando Silvestre et al. 34 utilizaram uma abordagem retroperitoneal minimamente invasiva semelhante à abordagem de Mayer para fusão intervertebral lombar anterior. Essa técnica é relatada por Silvestre et al. 34 como OLIF e acessa a superfície anterolateral do espaço discal antes do músculo psoas, que é mobilizado posteriormente. Após o preparo do espaço discal, o dispositivo intersomático é inserido em ângulo oblíquo e girado em posição lateral.

A evolução da técnica e da instrumentação proporcionou acesso adequado à coluna lombar, com baixos índices de complicações perioperatórias, tempos cirúrgicos menores, morbidade e mortalidade baixas, menor dor pós-operatória e retorno precoce às atividades diárias. As indicações incluem doenças degenerativas do disco, dor lombar discogênica, escoliose lombar degenerativa, espondilolistese de baixo grau, instabilidade lombar, revisão, estenose de canal branda a moderada, deformidade sagital, fraturas, tumores e doença em nível adjacente. 28

O dispositivo oblíquo pode elevar a altura do espaço intervertebral e expandir o tamanho do forame intervertebral, indiretamente levando à descompressão. 35 Quanto à acessibilidade anatômica, a técnica OLIF pode alcançar L1 a S1 usando um corredor oblíquo entre a aorta, a veia cava inferior e os músculos psoas para acesso ao espaço do disco.

Há espaço operatório adequado com o corredor oblíquo esquerdo dos níveis L2 a L5, com capacidade adicional de extensão durante a cirurgia com posicionamento lateral e retração do músculo psoas. A caixa torácica pode limitar o acesso a L1-L2, assim como a crista ilíaca e os vasos ilíacos podem limitar o acesso a L4-L5. 27 31

As vantagens são a menor invasividade da técnica operatória, o menor tempo cirúrgico e a menor chance de lesão do plexo lombar, permitindo acesso direto ao disco. A ausência de lesão da lâmina provoca menos danos e sangramento, menor taxa de lesão nervosa, acelera a recuperação pós-operatória, diminui a dor e o tempo de internação hospitalar e aumenta as taxas de fusão intersomática causada pela grande quantidade de remoção de disco, promovendo um grande área de contato com os platôs e adequada correção das deformidades. 6 36 37

Também há a possibilidade de abordar níveis mais superiores por meio do mesmo acesso sem ampliar a janela cirúrgica. As desvantagens incluem alterações anatômicas que limitam o procedimento e os possíveis riscos envolvidos, como disfunção simpática e lesão vascular. 21 31

Podemos observar que nas bifurcações aorta/cava/ilíaca superior (aquelas que ocorrem acima do terço superior de L4), os vasos ilíacos já estão bem abertos e seu trajeto é quase lateral ao nível do disco L4-5, o que causaria danos vasculares no suposto corredor de acesso à OLIF.

Considerando o relato acima, ao posicionar a lâmina inferior do afastador, deve-se evitar sua fixação na vértebra de L5 com parafuso específico. 6 21 31

A abordagem de acesso oblíquo tem várias limitações.

Existe o risco de estenose do canal causada pelo dispositivo intersomático no trajeto posteromedial, levando ao deslocamento do material discal ou ligamentar em direção ao canal central ou ao forame contralateral. Uma segunda limitação refere-se aos pacientes com espondilolistese de alto grau devido à ausência de “sobreposição” suficiente dos dois platôs vertebrais para acomodação do dispositivo intersomático em uma trajetória oblíqua.

O procedimento também pode ser influenciado pela estenose congênita do canal vertebral e pelas lesões que ocupam o espaço intervertebral, pela fusão espontânea do espaço intervertebral ou facetas posteriores e pelo formato do músculo psoas. Portanto, no lado côncavo dos pacientes com escoliose, o espaço entre os vasos e o músculo psoas diminui, o que não favorece a via OLIF.

A interiorização do sinal do psoas observado nas imagens axiais ao nível de L4-L5 se refere à presença do músculo psoas na área de entrada do anel lateral do disco vertebral. O espaço entre o músculo psoas e o músculo quadrado lombar é maior em alguns pacientes, o que pode levar à compreensão errada da distância entre a artéria e o músculo psoas. As diferentes posições influenciam o formato do músculo psoas. Em decúbito lateral direito, o músculo psoas maior esquerdo é afetado pela gravidade e fica próximo ao corpo vertebral. 6 31 38

## Abordagem Endoscópica

Para que uma abordagem À coluna seja considerada uma cirurgia totalmente endoscópica, precisa atender aos seguintes critérios: o uso de um endoscópio com canal funcional e sistema endoscópico, uma abordagem totalmente percutânea com uma pequena incisão, como uma punção na pele (“incisão por facada”) e técnica de portal único realizada sob irrigação constante com soro fisiológico. 39 40

Existem outros métodos de abordagem endoscópica da coluna vertebral, como endoscopia biportal, microendoscopia, epiduroscopia e cirurgia tubular assistida por vídeo. Entretanto, a cirurgia percutânea endoscópica ou completamente endoscópica da coluna ( Fig. 2 ) é a mais utilizada em nosso meio. 39 40

**Fig. 2:** Abordagem endoscópica transforaminal. (A) Localização da raiz do gânglio nervoso, (B) acesso direto ao abscesso no disco, (C) posicionamento da equipe, (D) visualização endoscópica da raiz nervosa.

Ao considerar a coluna lombar, as principais vias de acesso endoscópico podem ser divididas em acesso transforaminal e interlaminar. 41 42

## Abordagem Transforaminal

A abordagem transforaminal pode ser realizada com o paciente em decúbito ventral com flexão de quadris e joelhos sob anestesia local, sedação ou anestesia geral. Neste último caso, pode ser acompanhada por monitoramento neurofisiológico para que o cirurgião verifique a função da raiz emergente e estruturas próximas ao nível abordado. 41 42

Os princípios da via transforaminal endoscópica são obter o acesso mais próximo possível da doença discal e evitar a irritação da raiz emergente. Para atingir esses princípios, a foraminoplastia pode ser associada ao procedimento, que é a recalibração do forame intervertebral abordado utilizando meios para remover ou afinar as bordas ósseas. 42 43 Para tanto, podem ser usadas brocas (específicas de foraminoplastia ou não) ou referências percutâneas.

O ângulo de acesso e o ponto de acesso no forame são essenciais para o sucesso da técnica. Esses parâmetros podem ser ajustados de acordo com o tamanho corporal do paciente, o nível do disco e o sítio acometido a ser abordado. De modo geral, em níveis lombares inferiores (L4-L5 e L5-S1), utiliza-se uma distância da linha média de 10 a 12 cm e, em níveis superiores, a abordagem é progressivamente mais próxima da linha média devido à topografia renal.

Após a incisão posterolateral, um fio-guia é inserido para atingir o espaço do triângulo de Kambin. Este triângulo de segurança de abordagem é definido como o espaço entre a raiz emergente lateralmente, medialmente a dura-máter, e a base, a porção superior do pedículo da vértebra inferior.

A via de acesso é dilatada com afastadores rombos progressivos até a colocação do canal de trabalho. Depois do posicionamento seguro do canal, o endoscópio é inserido. A forma ideal de garantir o posicionamento correto é a visualização adequada das estruturas que compõem o forame intervertebral para aumentar a segurança da cirurgia. 42 43

Existem algumas divergências na literatura quanto à abordagem do disco. A princípio, a abordagem endoscópica transforaminal era realizada a partir da porção central do disco e progredia em direção à parte externa do disco, técnica conhecida como *inside-out* .

Com a evolução das técnicas cirúrgicas e tecnologias de equipamentos para doenças discais, surgiu a técnica de abordagem externa, conhecida como *outside-in* . 42 43 44

Recentemente, a abordagem transforaminal de doenças ósseas do forame, sem necessidade de acesso ao disco, a técnica *outside-out* corresponde à utilizada para descompressão óssea do forame. Essa abordagem é associada ao risco de dor residual devido à manipulação radicular pelo procedimento, principalmente durante a curva de aprendizado do cirurgião. No entanto, as complicações neurológicas são menores nas técnicas endoscópicas percutâneas em comparação à abordagem aberta. 45

## Abordagem Interlaminar

Como a endoscopia transforaminal, a abordagem interlaminar pode ser realizada sob sedação ou anestesia local ou geral. O ponto alvo da abordagem é a borda lateral da janela interlaminar. 41 43 Essa janela costuma ser mais proeminente em níveis mais caudais e diminui de maneira progressiva em sentido cefálico. Assim, L5-S1 é o nível com maior janela interlaminar. Inicialmente, a via de acesso interlaminar exigia janelas de bom tamanho para garantir uma abordagem segura.

A evolução da técnica cirúrgica e dos equipamentos de descompressão óssea possibilitou a abordagem de janelas interlaminares ainda menores após a descompressão óssea adequada. 43 46 A abordagem interlaminar pode começar com um fio-guia ou mesmo com a abordagem direta com o afastador rombo devido ao risco de introdução inadvertida do fio-guia no canal vertebral através do ligamento amarelo.

Após o afastador, a cânula de trabalho é posicionada com o bisel apoiado na porção mais lateral da janela. A seguir, o endoscópio é introduzido para garantir a visualização do ligamento amarelo. 41 46 Este ligamento pode ser removido com pinça e tesoura apropriadas, permitindo a visualização do espaço peridural.

Após a flavectomia, o campo cirúrgico é preparado com descompressão óssea mais eficaz e/ou remoção de partes moles até que seja possível visualizar e dissecar as estruturas nervosas. 41 46 Esta via permite o tratamento de doença compressiva discal ou óssea, dependendo da origem dos sintomas. Ao final da cirurgia, a descompressão pode ser confirmada com sonda romba através do canal de trabalho e visualização de estruturas livres e pulsáteis com o fluxo sérico. 41 46

## Considerações Finais

As diversas vias de acesso e abordagens cirúrgicas aqui descritas continuam sendo objetos de estudo, gerando dezenas de publicações anualmente. Não há consenso sobre a melhor abordagem para cada doença. Porém, uma tendência busca redução dos danos teciduais, principalmente musculares, eficácia na descompressão de estruturas nervosas e maior segurança com minimização dos riscos de danos neurológicos e infecções pós-operatórias. Além disso, os avanços nas técnicas de imagem e ampliação permitem acessos menores, mais eficazes e mais seguros.
